# *In Vitro* Toxic Effects of Zinc Oxide Nanoparticles on Rat Adipose Tissue-Derived Mesenchymal Stem Cells

**DOI:** 10.22074/cellj.2015.2

**Published:** 2015-10-07

**Authors:** Mahmoud Orazizadeh, Ali Khodadadi, Vahid Bayati, Sadegh Saremy, Maryam Farasat, Layasadat Khorsandi

**Affiliations:** 1 Cell and Molecular Research Center, Faculty of Medicine, Ahvaz Jundishapur University of Medical Sciences, Ahvaz, Iran; 2 Department of Anatomical Sciences, Faculty of Medicine, Ahvaz Jundishapur University of Medical Sciences, Ahvaz, Iran; 3 Department of Immunology and Cancer, Petroleum Pollutants Research Center, Ahvaz Jundishapur University of Medical Sciences, Ahvaz, Iran

**Keywords:** Nanoparticles, Mesenchymal Stromal Cells, Apoptosis

## Abstract

**Objective:**

Zinc oxide nanoparticles (ZnO-NPs) are increasingly used in sunscreens, bio- sensors, food additives, pigments, manufacture of rubber products, and electronic materi- als. There are several studies about the effects of NPs on dermal fibroblast or keratino- cytes, but very little attention has been directed towards adipose-derived mesenchymal stem cells (ASCs). A previous study has revealed that ZnO-NPs restricted the migration capability of ASCs. However, the potential toxicity of these NPs on ASCs is not well un- derstood. This study intends to evaluate the effects of ZnO-NPs on subcutaneous ASCs.

**Materials and Methods:**

In this experimental study, In order to assess toxicity, we ex- posed rat ASCs to ZnO-NPs at concentrations of 10, 50, and 100 µg/ml for 48 hours. Tox- icity was evaluated by cell morphology changes, cell viability assay, as well as apoptosis and necrosis detection.

**Results:**

ZnO-NPs concentration dependently reduced the survival rates of ASCs as re- vealed by the trypan blue exclusion and 3-(4,5-Dimethylthiazol-2-yl)-2,5-diphenyltetrazo- lium-bromide (MTT) tests. ZnO-NPs, at concentrations of 10 and 50 µg/ml, induced a significant increase in apoptotic indices as shown by the annexin V test. The concentration of 10 µg/ml of ZnO-NPs was more toxic.

**Conclusion:**

Lower concentrations of ZnO-NPs have toxic and apoptotic effects on subcutaneous ASCs. We recommend that ZnO-NPs be used with caution if there is a dermatological problem.

## Introduction

Metal nanoparticles (NPs) and their oxides have a considerable number of present and future applications in the medical and industrial fields. The smaller size and unique properties of NPs has substantially improved their application (1-3). One of the most popular industrial applications of NPs is sunscreens (4). A major component of sunscreens, zinc oxide NPs (ZnO-NPs) can effectively absorb ultraviolet light (5). ZnO-NPs are also used in several products such as biosensors, food additives, pigments, manufacture of rubber products, and electronic materials (6). Despite their wide use, very little data on their toxic effects are available. *In vivo* studies have demonstrated that ZnO-NPs show greater adverse effects when compared to normal-scale particles (7, 8).

DeLouise (9) reported that ZnO-NPs were nontoxic for cultured human dermal fibroblasts. Other reports also suggested that ZnO-NPs were toxic for dermal fibroblasts (10), neuroblastoma cells (11) and vascular endothelial cells (12). However, toxicological studies have not provided a complete understanding of the potential toxicity of ZnO-NPs.

Mesenchymal stem cells within the stromalvascular fraction of subcutaneous adipose tissue, adipose-derived mesenchymal stem cells (ASCs), display multi-lineage developmental plasticity and secrete various growth factors that control and manage damaged neighboring cells. The synthesis and secretion of growth factors are essential functions of ASCs. Regeneration diversity of ASCs has been shown in skin (13). Conditioned medium from ASCs can stimulate both collagen synthesis and migration of dermal fibroblasts, which improves wrinkles and accelerates wound healing in animal models (14). ASCs also protect dermal fibroblasts from oxidative stress induced by chemicals and Ultraviolet irradiation (15).

There are several studies of the NPs effects on dermal fibroblast or keratinocytes, but very little attention has been directed towards ASCs. In present study, we have investigated the effects of ZnO-NPs on rat subcutaneous ASCs.

## Materials and Methods

### Cell culture

This experimental study was approved by the Ethics Committee of Ahvaz Jundishapur University of Medical Sciences. For this experiment, we used 12 female Wistar rats. Subcutaneous adipose tissues were removed from the rats under sterile conditions and cut into small pieces. The removed tissues were incubated in order seeding the cells in 25 cm^2^ flasks that contained Dulbecco’s modified eagle’s medium (DMEM, Invitrogen, Carlsbad, CA) and 1.0 mg/ml of collagenase type IA (C9891, Sigma, USA). Incubations were performed for 15 minutes at 37˚C in a water bath where the flasks were shaken at a speed of 120 cycles/minute. After 10 and 15 minutes, respectively, the flasks were vigorously mixed for 10 seconds, after which the contents of the flasks were filtered through a nylon screen (250 μm pore size) to collect any remaining and non-disintegrated tissue. The cell suspension was subsequently centrifuged at approximately 300 g for 3 minutes. When a homogenous cell suspension was achieved, the suspension cells were centrifuged at 1200 rpm for 7 minutes and 3 ml of culture medium was added to the cell pellet and pipetted. The cells were cultured in 25 cm^2^ flasks with 5 ml DMEM and maintained at 37˚C in a humidified atmosphere of 5% CO_2_. The culture media were changed every three days. Cells were passaged after they reached approximately 75% confluency. The mesenchymal population was isolated on the basis of its ability to adhere to the bottom of the flask (16).

### Characterization of adipose-derived mesenchymal stem cells

We used flow cytometry to analyze cell surface marker expressions by the ASCs culture prior to ZnO-NPs treatment. The cells were characterized according to a set of cell surface markers characteristic for ASCs that included cluster of differentiation (CD) CD73 (sc-14682, Santa Cruz Biotechnology Inc., USA), CD105 (sc-20632, Santa Cruz Biotechnology Inc., USA), CD45 (sc-25590, Santa Cruz Biotechnology Inc., USA) and CD34 (sc-7045, Santa Cruz Biotechnology Inc., USA).The differentiation potentials of the ASCs were checked in specific media. For adipocyte differentiation, cells were cultured in 1 μmol/l dexamethasone (D4902, Sigma, USA), 60 μmol/l indomethacin (I7378, Sigma, USA), and 450 μl 3-isobutyl- 1-methylxanthine (I5879, Sigma, USA). Adipocytes were characterized by oil red-O (O0652, Sigma, USA) staining under a light microscope. For differentiation into osteoblasts, culture medium was supplemented with 0.1 μmol/l dexamethasone, 10 mmol/l β-glycerophosphate (G9422, Sigma, USA) and 60 mmol/l ascorbate (A0157, Sigma, USA). Osteoblasts were characterized by alizarin red (A5533, Sigma, USA) staining and microscopic examination (16).

### Experimental design

Passage 3 ASCs, after characterization, were used in this experiment. For each treatment at least 16 flasks of culture cells were used [8 flasks for trypan blue and the 3-(4,5-Dimethylthiazol-2-yl)-2,5-diphenyltetrazolium- bromide (MTT) assay, 8 flasks for annexin/propidium iodide (PI)]. These cultures were obtained from 12 female adult Wistar rats. The control group consisted of untreated cells. Experimental groups were treated with 10, 50, and 100 μg/ ml of ZnO-NPs, respectively, for 24, 48 and 72 hours. All three concentrations were treated at all three time periods. ZnO-NPs (100 mg/ml, 677450, Sigma, USA) was prepared in the culture medium and dispersed for 10 minutes by using a sonicator. The stock solution of ZnO-NPs was kept at 4˚C and used within one week for the experiments. Just prior to use, the stock solution was diluted in culture medium and prepared by ultrasonication (Solid State/Ultrasonic FS-14, Fisher Scientific) for 15 minutes to prevent aggregation. To ensure nonaggregation of ZnO-NPs, the time interval from preparation to use was strictly limited to less than 20 minutes. In addition, 20 minutes after preparation, the particle size of ZnO-NPs was analyzed by atomic force microscopy (AFM).

### Cell morphology

Morphological observations using an inverted light microscope (Olympus, IX71) were performed. Digital micrographs were documented by a digital camera (Olympus E620-SLR) coupled to the microscope.

### Cell viability and proliferation

Cell viability was measured by trypan blue (9). Trypsin/Ethylenediaminetetraacetic acid (EDTA, T4049, Sigma, USA) cell suspensions of the culture ASCs were used to measure cell viability. A total of 20 μl of the cell suspension (500 cells/μl) and 20 μl of trypan blue reagent (T6146, Sigma, USA) were mixed and incubated at room temperature for 5 minutes. Viable cells were counted using a Neubauer hemocytometer under a light microscope (Olympus IX71). MTT assays were also used to determine the effect of ZnO-NPs on cell viability and proliferation. Briefly, cells were maintained with culture media for 24, 48 and 72 hours in 24- well plates. MTT (M2128, Sigma, USA) was then added to each well (0.5 mg/ml) and cells were further incubated for 4 hours at 37˚C. We removed the supernatants and 700 μl of dimethyl sulfoxide (DMSO, D2650, Sigma, USA) was added to each well to dissolve the formazan product. A 680 Microplate reader (BioRad, Hercules, CA) was used to measure absorbance at 540 nm. MTT assay values were expressed as the percentage of corresponding average values in control cultures. Absorbance is in proportion to the number of living cells in a sample. Thus, the MTT assay indicates the extent of cell proliferation (17).

### Annexin V-FITC/propidium iodide apoptosis assay

ASCs were placed in a six-well culture plate and treated with 10, 50 and 100 μg/ml of ZnONPs for 48 hours. Normal, apoptotic, and necrotic cells were distinguished using an Annexin V-fluorescein isothiocyanate (FITC)/PI Assay Kit (V13242, Molecular Probes/Invitrogen, Carlsbad, CA, USA) according to the manufacturer’s instructions. Briefly, after the incubation period, the cells (1.0×10^6^) were washed in cold phosphate-buffered salin (PBS). The washed cells were re-centrifuged. After the supernatant was discarded, cells were resuspended in 1X annexin-binding buffer. We added 25 μl of annexin V conjugate and 2 μl of the 100 μg/ ml PI working solution to each 100 μl of cell suspension. The cells were incubated at room temperature for 15 minutes after which they were washed with 1X annexin-binding buffer and deposited onto slides. Fluorescence was observed by using appropriate filters. The cells were separated into three groups: live, apoptotic, and necrotic. Weak annexin V staining was observed only on the cellular membrane of live cells. Apoptotic cells showed higher degrees of cell surface labeling. In necrotic cells both membrane stained by annexin V (green) and strong nuclear staining from PI (red) was observed.

We calculated the apoptotic index (AI) and necrotic index (NI) by dividing the number of apoptotic or necrotic cells in a random microscopic field by the total number of cells in that field, multiplied by 100. The AIs and NIs of 10 random fields were evaluated and the mean AI and NI of each case were calculated.

### Statistical analysis

Comparisons of multiple (>3) group means (AI, trypan blue and MTT assay) were performed using one-way analyses of variance and post hoc procedures based on the Newman-Keuls tests. The student’s t tests were used for comparisons of two group means. A P value less than 0.05 was considered statistically significant.

## Results

### Atomic force microscopy

**Fig.1:**
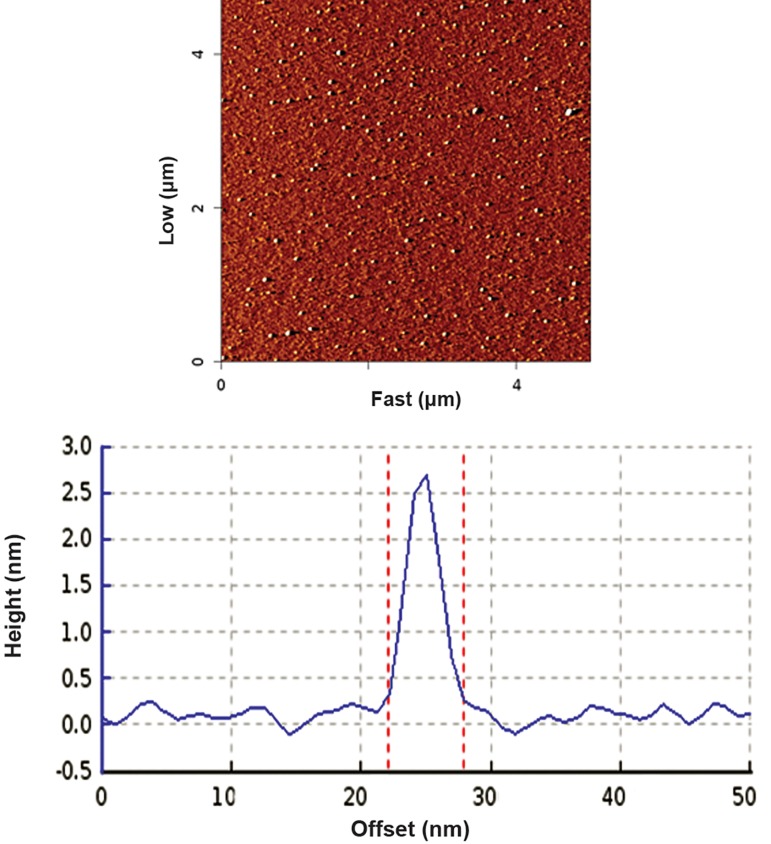
Atomic force microscopy (AFM) image of nanoparticles (NPs) showed distinct spherical particles at size ranges between 20 and 30 nm.

AFM showed the size and morphology of the synthesized particles. The complexes appeared spherical with a mean size of 20-30 nm (Fig .1).

### Cell culture characterization

**Fig.2:**
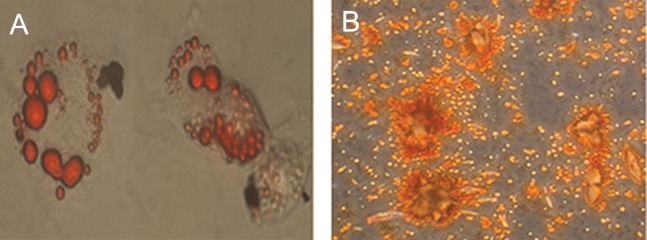
Characteristics of adipose-derived mesenchymal stem cells (ASCs) at passage 3. A. Potential differentiation of ASCs into osteogenic (alizarin red staining, magnification: ×1000) and B. Potential differentiation of ASCs into adipogenic (oil red O staining, magnification: ×400).

In culture, the ASCs appeared to have a spindle shape. They were harvested at passage 3 and characterized as mesenchymal stem cells according to flow cytometry analysis and differentiation potential. Cell surface markers detected by flow cytometry showed that ASCs highly expressed CD105 (96%) and CD73 (83%), whereas no expressions of CD34 and CD45 were detected. As expected and previously described, culture in the appropriate media showed that isolated ASCs were capable of *in vitro* differentiation into osteoblasts and adipocytes (Fig .2).

### Effects of zinc oxide nanoparticles on cellular morphology

**Fig.3:**
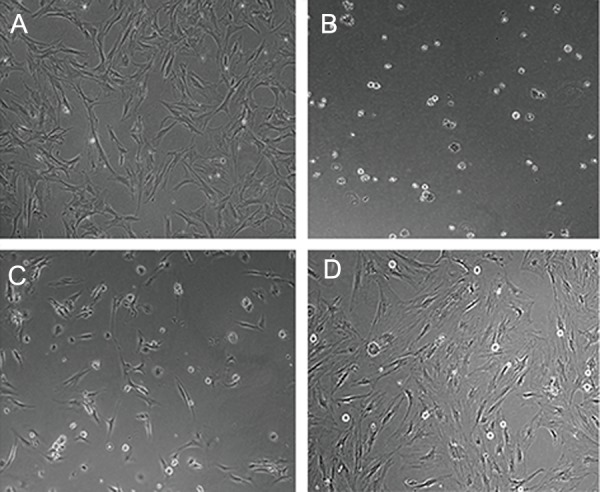
Zinc oxide nanoparticles (ZnO-NPs) effects on adipose-derived mesenchymal stem cell (ASCs) morphology. A. Control group cells have a fibroblast-like morphology, B. Treatment with 10 μg/ml of ZnO-NPs. Numerous round shape cells are observed, C. Treatment with 50 μg/ml of ZnO-NPs. The round morphology are decreased and D. Treatment with 100 μg/ml of ZnO-NPs. The majority of cells exhibit a fibroblast-like morphology (magnification: ×250).

Untreated ASCs (control) exhibited an elongated spindle morphology. Cells treated with 10 μg/ml of ZnO-NPs showed morphological changes of cell damage (Fig .3A). At this concentration the majority of ASCs had a round shape with a shrunken morphology (Fig .3B). At 50 μg/ml, the numbers of cells with round shape morphology were less than cells exposed to 10 μg/ml (Fig .3C). At 100 μg/ml ZnO-NPs, the majority of ASCs had normal spindle morphology (Fig .3D).

### Cell viability and proliferation

**Fig.4:**
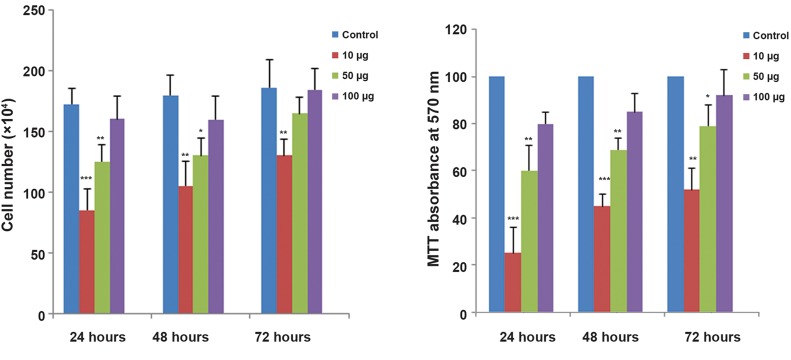
A. Effect of zinc oxide nanoparticles (ZnO-NPs) on adipose-derived mesenchymal stem cell (ASCs) viability by the trypan blue and B. 3-(4,5-Dimethylthiazol-2-yl)-2,5-diphenyltetrazolium-bromide (MTT) assays. Values are expressed as mean ± standard deviation (SD). *; P<0.05, **; P<0.01 and ***, P<0.001.

Trypan blue exclusion showed that ZnO-NPs induced a decrease in the number of viable cells in a concentration-dependent manner. As shown in figure 4, ZnO-NPs at a concentration of 10 μg/ml for 24, 48 and 72 hours induced a strong decline in the number of viable cells (P<0.001). ASCs treated with 50 μg/ml of ZnO-NPs for 24 and 48 hours showed significant decrease in viable cell number (P<0.01). No significant changes in the number of viable cells were observed following exposure of ASCs to 100 μg/ ml ZnO-NPs at 24, 48 and 72 hours.

MTT assays showed that exposure of ASCs to 10 and 50 μg/ml of ZnO-NPs induced a significant decrease in cell viability (P<0.01). No significant changes in cell viability were observed at 100 μg/ml of ZnO-NPs (P>0.05). The MTT proliferation assay also showed that ZnO-NPs had a concentration-dependent effect on ASC cell growth (Figes.4, 5).

**Fig.5:**
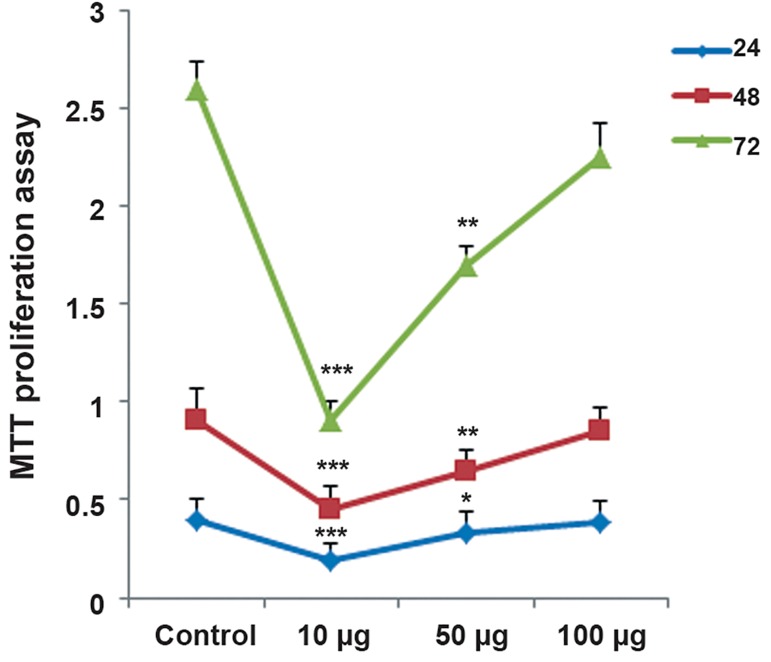
Effect of zinc oxide nanoparticles (ZnO-NPs) on proliferation of adipose-derived mesenchymal stem cells (ASCs) by trypan blue and the 3-(4,5-Dimethylthiazol-2-yl)-2,5-diphenyltetrazolium-bromide (MTT) assay. Values are expressed as mean ± standard deviation (SD). *; P<0.05, **; P<0.01 and ***; P<0.001.

### Annexin V-FITC/propidium iodide apoptosis assay

A few necrotic cells were observed in the control group. Treatment with 10 μg/ml of ZnO-NPs caused a large number of ASCs to undergo apoptosis and necrosis. The numbers of both apoptotic and necrotic cells at 50 μg/ml significantly increased compared to the control group (P<0.01). AI and NI significantly decreased compared to 10 μg/ml treated cells (P<0.01). No significant changes in the number of apoptotic cells were observed at exposure of ASCs to 100 μg/ ml (P>0.05). AI and NI significantly decreased compared to 10 μg/ml treated cells (Figes.6, 7).

**Fig.6:**
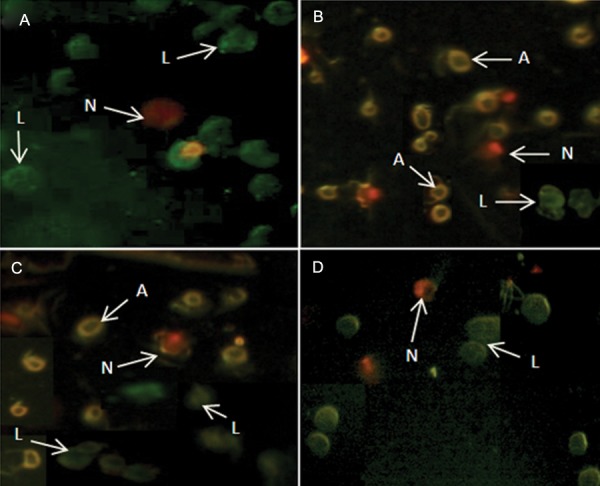
Fluorescent microscopy of annexin Vfluorescein isothiocyanate (FITC)/propidium iodide (PI) stained adipose-derived mesenchymal stem cells (ASCs) from control and experimental groups. A. Control group, B. Treatment with 10 μg/ml of zinc oxide nanoparticles (ZnO-NPs), C. Treatment with 50 μg/ml of ZnO-NPs and D. Treatment with 100 μg/ml of ZnO-NPs. A; Apoptosis (cell membrane is strongly stained), N; Necrosis (nucleus is red) and L; Live (cells show green stain, magnification: ×250).

**Fig.7:**
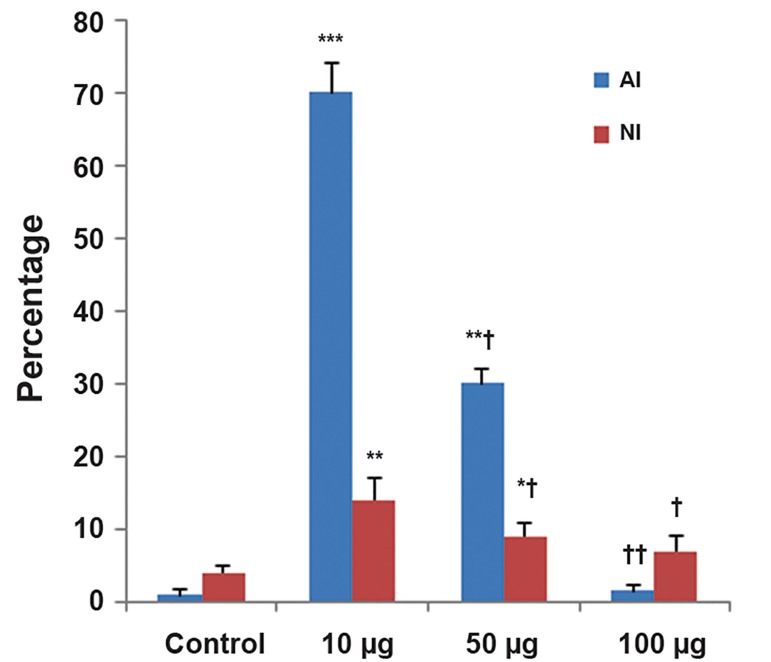
Apoptotic index (AI) and necrotic index (NI) in control and experimental groups. Values are expressed as mean ± standard deviation (SD) for eight rats. *; P<0.05, **; P<0.01, ***; P<0.001, †; P<0.01, ††; P<0.001. * and † symbols respectively indicate comparison to control and 10 μg/ml of zinc oxide nanoparticles (ZnO-NPs) treated cells.

## Discussion

Our results have demonstrated that ZnO-NPs had concentration-dependent toxic effects on ASCs, induced loss of viability, apoptosis induction and morphological changes. To our knowledge this study is the first to demonstrate the cytotoxic effects of NPs on subcutaneous ASCs. A previous study has shown that ZnO-NPs impaired migration capability of ASCs (18). Numerous researchers reported that ZnO-NPs caused cytotoxic effects to various cell types such as osteoblast cancer cells (19), human bronchial epithelial cells (BEAS-2B) (20), human kidney cells (21), human alveolar adenocarcinoma cells (22) and human hepatocytes (23).

In the present study, ASCs treated with a lower concentration of ZnO-NPs appeared to be strongly damaged as evidenced by their round morphology and detachment from the substrate which indicated almost complete cell destruction. This typical change in morphology reflected drug toxicity.

To confirm that these signs of morphology were followed by a reduction in ASCs growth after treatment with ZnO-NPs, we measured MTT activity. We found a concentration-dependent decrease in ASC viability. Similar results were obtained with trypan blue exclusion. Pasupuleti et al. (24) administered 5, 50, 300, 1000 and 2000 mg/kg body weight of nanoand micro-sized ZnO to Sprague Dawley rats. They reported that the incidences of toxicity in the liver, pancreas, heart and stomach were higher at lower doses of ZnO-NPs compared to higher doses.

However, Talebi et al. (25) demonstrated that ZnO-NPs at 50 and 300 mg/kg body weight had toxic effects on mouse spermatogenesis while no significant changes in spermatogenesis were observed at the 5 mg/kg dose.

In the present study, ZnO-NPs inhibited cell proliferation in a concentration-dependent manner. Taccola et al. (26) reported that ZnO-NPs showed cytotoxic effects on rapidly proliferating benign and malignant cells. To confirm that the reduction of viability was due to apoptosis or necrosis, the cells were assayed with the annexin V-FITC/ PI apoptosis assay. Annexin V binds to phosphatidylserine that is translocated from the inner to the outer leaflet when apoptosis occurs in a cell (27, 28). PI is used as a DNA stain, which is excluded by the cell membrane of living cells. Exposure to PI without prior permeabilization is used to distinguish necrotic cells from apoptotic and living cells (29). In the experimental group, especially at the 5 mg/kg dose, the percentage of annexin V-positive cells significantly increased. The NI at lower concentrations also significantly increased. Our results revealed that apoptosis, not necrosis, was the predominant mechanism in ZnO-NPs-induced cytotoxicity.

Wilhelmi et al. (30) reported that ZnO-NPs induced necrosis and apoptosis in macrophages. Meyer et al. (10) reported that ZnO-NPs induced apoptosis in human dermal fibroblasts. Sharma et al. (31) also stated that ZnO-NPs, even at low concentrations, possessed a genotoxic potential in human epidermal cells. On the other hand, ASCs had beneficial effects on skin disease (32). Chavez- Munoz et al. (33) have stated that adipose tissue is a suitable source for keratinocytes. According to recent studies, ASCs have wound-healing and antioxidant properties. ASCs and their secretory factors show promise for use in skin repair and regeneration. Thus, reduction in ASCs viability may suppress wound healing as factors secreted by ASCs can induce dermal fibroblast proliferation, migration and extracellular matrix secretion (33). Additionally, Kocbek et al. (34) have demonstrated that exposure to ZnO-NPs can induce adverse effects on human keratinocytes *in vitro*. On the other hand, Hackenberg et al. (35) showed that titanium dioxide NPs and ZnO-NPs impaired the migration capability of ASCs. They concluded that restricted migration might critically influence wound healing capacity.

The amount of time and exposure concentration to ZnO-NPs are important factors that affect cytotoxicity (36). In this study, ASCs have been exposed to ZnO-NPs for 24, 48 and 72 hours. Mesenchymal stem cells (MSCs) have a limited life span in cultures and undergo senescence as indicated by loss of proliferation and altered morphology (37). Proliferation capacities of MSCs derived from BM-MSCs, UCB-MSCs and ASCs have been analyzed by Kern et al. (18). They reported that ASCs possessed the lowest population doubling numbers after passage 3. Additionally, Rubio et al. (38) stated that after long-term *in vitro* expansion, the ASCs populations could immortalize and transform spontaneously. Thus, in the current study, short-term exposure to ZnO-NPs was considered. Hackenberg et al. (39) exposed Ag-NPs to human MSCs (hMSCs) for 1, 3 and 24 hours. Cytotoxic effects were reported at all test exposure periods.

The exact mechanism of ZnO-NPs toxicity on ASCs was not determined in the current study. The reason for significant toxicity at lower concentrations was not clear. It has been demonstrated that NPs at high concentrations tend to cluster and form aggregates often larger than 100 nm. Thus, absorption of NPs by cells might be limited at high concentrations (40, 41). One possibility was that more ZnO-NPs at lower concentrations might be absorbed which could stimulate various signaling pathways involved in apoptosis or necrosis. Additionally, high concentrations of ZnO-NPs might have stimulated anti-apoptotic molecules inside ASCs. Future experiments are needed to clarify these hypotheses.

## Conclusion

The present study has demonstrated that ZnONPs at lower concentrations have toxic and apoptotic effects on subcutaneous rat ASCs. This data provides a stimulus for true clinical studies. Further experiments are needed to clarify the mechanisms of ZnO-NPs induced cytotoxicity in MSCs.
